# Medical Students' Perception of Virtual Simulation-Based Learning in Pharmacology

**DOI:** 10.7759/cureus.33261

**Published:** 2023-01-02

**Authors:** Salah Eldin A Abdel Haleem, Aimun A Ahmed, Haitham El Bingawi, Aziz Elswhimy

**Affiliations:** 1 Department of Pharmacology, Faculty of Medicine University of Bahri, Khartoum, SDN; 2 Department of Pharmacology, Faculty of Medicine Albaha University, Albaha, SAU; 3 Department of Pharmacology, Faculty of Pharmacy Omdurman Islamic University, Khartoum, SDN; 4 Department of Medicine, Sultan Qaboos University, Muscat, OMN; 5 Department of Internal Medicine, Faculty of Medicine Albaha University, Albaha, SAU

**Keywords:** learning experience, teaching methods, virtual simulations, educational technology, pharmacology education

## Abstract

Virtual simulation-based learning has opened a vista for surmounting ethical issues with the use of animals in compliance with one of the ‘3Rs’ in ethical principles for animal use, which is ‘replacement.’ It's effective in terms of time, place, and cost. For instance, the time for drug application in cancer models would be less with virtual simulations, and the cost of maintenance and update of the software is less than that of breeding and feeding experimental animals. This paper examines the effects of utilizing a virtual computer tool simulating real pharmacology laboratory equipment in the second semester of a large-scale basic medical course. We looked at the theories of education and instructional designs and used them to develop a virtual computer lab that could help our students meet the intended learning outcomes. We analyzed, developed, implemented, and finally evaluated the students' reactions (at the Kirkpatrick level) using a self-administered questionnaire with responses on a three-point Likert scale. Feedback was obtained from 60 out of 82 (73.2 %) level 4 medical undergraduate students of both sexes, 39/60 (65%) were from the male section of the college. Sixty percent of the students admitted that the software is simple. Sixty percent agreed that it was good. Fifty-seven percent denied previous exposure to the simulation lab. Fifty-two percent reported that the practical lab's content was good, 53.3% rated the achievement of the practical objectives as good, 48.3% rated the practical enforcement of theoretical knowledge as good, 61.7% estimated getting realistic results, 48.3% agreed that the simulation lab encourages formulating a live experiment to test the hypothesis, and 51.7 % decided that the time framework was long. Thirty-eight percent appreciated the learning experience, and 45% felt that it should be repeated elsewhere. Students from the female section opted to record different determinations. The experience of using the virtual computer lab as part of the teaching program in pharmacology confirms the educational value of simulation. By adding a flexible reliable teaching method, we believe it served as a valuable tool for assisting teaching and learning in our context. Moreover, it is perceived as favorable by a good number of our students.

## Introduction

Practical sessions are a robust method that helps students develop an intuitive understanding of concepts and to liven up the knowledge provided in lectures. Be that as it may, how much students learn from these sessions depends on the way they are conducted [1]. Laboratory classes are primarily concerned with illustrating and reinforcing theoretical concepts taught elsewhere [2]. Virtual labs can be an attractive choice, and sometimes the only choice, in terms of cost, time, space, and safety. They pose little ethical concerns for traditional lab use of experimental animals [3]. Numeric evidence is well-established that computer-based simulations can be used productively in lieu of real equipment [4]. In a virtual setting, students thrive on a learning experience rich in visual cues, instant feedback, and self-pacing options for repeating a unit or moving quickly ahead. Be that as it may, missing the motivation of the tools e.g., for animals’ restraint and dissection, the sights and smells, and the surprising outcomes of experimentation experienced in a wet hands-on pharmacology lab setting negatively impacts this memorable learning experience, especially if you need to track pharmacology experiments involving behavioral or psychological issues [3]. Students who used the simulated equipment outperformed their counterparts, who used real laboratory equipment both on a conceptual survey of the domain and in the coordinated tasks [4]. This discrepancy might largely be attributed to a lack of experience rather than a weak point in live animal studies. A review of the literature on the differences in gender gain, between traditional lecture and interactive engagement, is large enough that it could impact the results of studies comparing the effectiveness of different teaching methods [5]. The difference between male and female responses is most likely due to the combination of many small factors rather than any one factor that can easily be modified [5].

The study justification

The present work explores the utilization of computer-based simulations in the field of pharmacology experiments in medical education wherein students are reluctant to handle animal-based wet labs. The intention was directed to validate these alternative methods in terms of student perception of their reliability, reality, learning experience, and learning outcomes.

## Materials and methods

The University of Strathclyde UK simulated organ bath software (ObSim, version 2.8) with the capacity to simulate the responses of in-vitro isolated tissue preparations was utilized in conducting the pharmacology practical sessions that cover autonomic drugs in the Principles of Disease II Module for level 4 medical students at Al Baha University, KSA. Instructors have designed effective Practical Handout Guidelines that guided them throughout practical sessions. The observational, analytical descriptive study was conducted during the academic years 2017/2018 and 2018/2019. A convenient sample size was used wherein all the students of these academic years who attended the target labs and consented to participate in the post-activity evaluation were included. A total of 60 out of 89 were enrolled in the study. A three-domain data collection tool was used to collect: 1. demographic information, 2. facilities and software evaluation, and 3. content evaluation regarding its capacity to 1. achieve the intended learning objectives (ILOs), 2. generate realistic results covering the disciplinary pharmacological parameters, 3. encourage the formulation of wet hand-on experiments, and 4. provision of time-effective lab procedures. Instructors presented demonstrations during the lab. sessions. Students were told to follow the same procedure when it came to each demonstration. Immediately after each demonstration and practical lab conduction, students were asked to answer six feedback-structured questions and an open-end reflection response. Students were asked to report the numeric outcome of a practical lab by responding to a pre-designed lab report and by attaching their tissue-screening graph determinations. Reports were designed to probe students’ understanding of the ‘pharmacology’ underlying each demonstration and spanned the full range of possible outcomes. Students’ Lab Evaluation Tool sheets were collected right after the practical lab while students had a day to produce and deliver lab reports. During the process of results coding, regarding (1) the sex of the students and (2) coded for male and female, respectively. Regarding domain 1 questions, 1, 2, and 3 coded for excellent, good, and bad, respectively. Regarding domain 2 questions, 1, 2, and 3 coded for yes, no, and I don’t know, respectively. The last question of domain 2 regarding the time of the practical session, 1, 2, and 3 coded for long, suitable, and short, respectively. SPSS software (IBM Corp., Armonk, NY) was used for data coding, entering, analysis, and categorizing appropriate figures and tables as results of descriptive statistics of a modified three-point Likert scale to obtain the study results. Standard Likert scale surveys are primarily created on a scale of 5 or 7, having a neutral position at the center. At times, a simple yes or no could be sufficient when target audiences need to be surveyed about their perceptions and opinions. In the present study, we needed a response with a neutral position for the questions of domain 1 regarding software use while we needed our response with a clear opinion without being neutral for the questions of domain 2 regarding the lab. While the analysis presented represents results pooled from all classes, each class was first analyzed separately. Results were combined after establishing identical trends in each.

## Results

**Figure 1:**
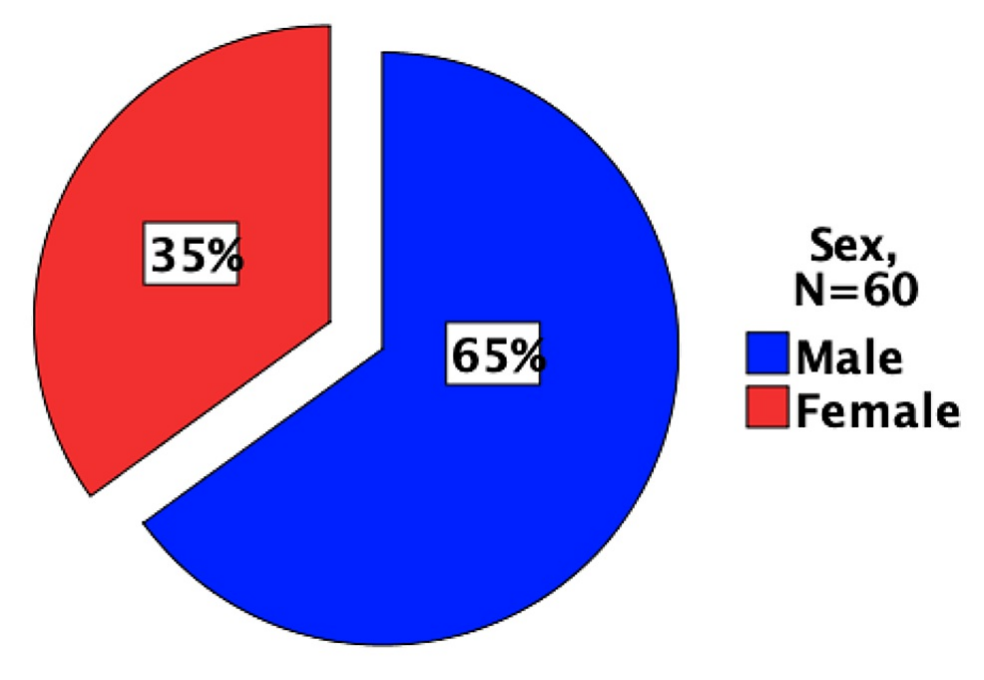
Gender frequency distribution of the study participants showing that 65% were male medical students

Demographic results assign 65% of the level 4 medical undergraduate student participants to the male gender (Figure 1).

**Figure 2:**
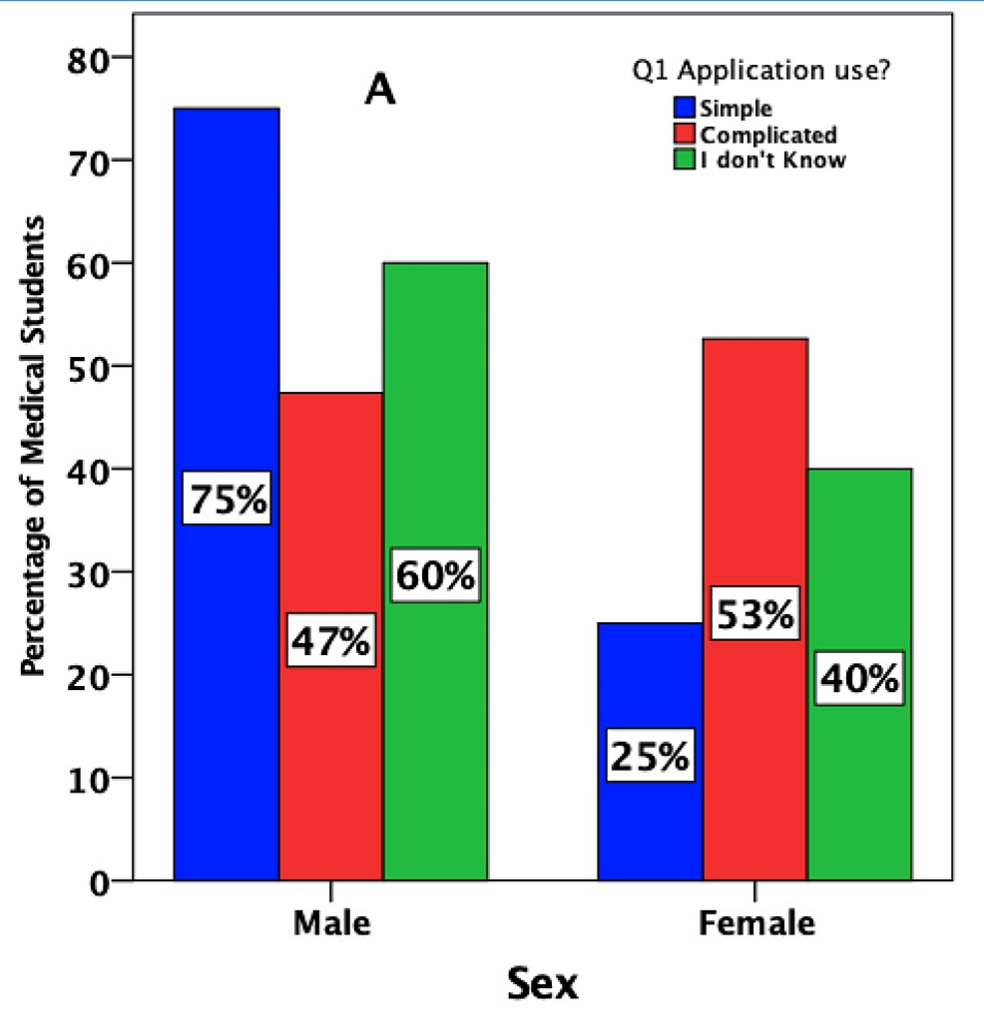
Medical students' estimates of the ease of handling and quality of the learning experience regarding pharmacology virtual lab simulation software

**Figure 3:**
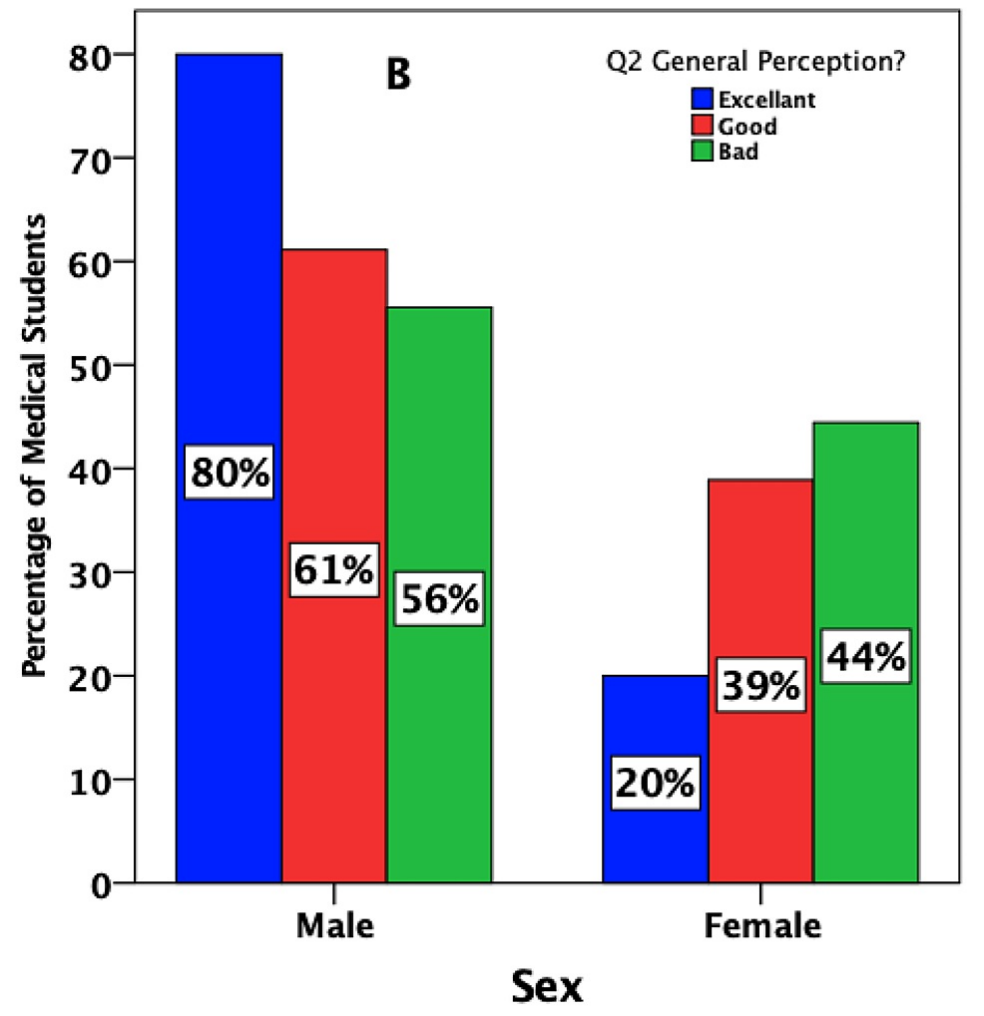
Medical students’ estimates of the ease of handling and quality of the learning experience regarding the pharmacology virtual lab simulation software

Two-thirds (75%) of males reported that the handling of the tool is simple, with the provision of an excellent learning experience (80%). Be that as it may, the perception of female students was different (Figures 2, 3).

Regarding the scientific content delivered by the practical lab, half of the students (52%) reported that it was good, 53.3% rated the achievement of the practical objectives as good, 48.3% rated the practical enforcement of theoretical knowledge as good, 61.7% reported getting realistic results, 48.3% agreed that the simulation lab encourages formulating a live experiment to test the hypothesis, 51.7% decided that the time framework was long. Thirty-eight percent appreciated the learning experience, and 45% felt that it should be repeated elsewhere in collateral disciplines. Students from the female section opted to record different, but generally consistent, determinations. All these results are shown in Table 1.

**Table 1:** Response of medical students to the questions regarding the scientific content delivered by a simulated pharmacology practical, n=60

#	Question	Kirkpatrick Level	Sex	Response (Frequency/%)		
				Excellent	Good	Bad
Domain 1: Software						
1	Please grade the adequacy of the simulated practical lab. in the delivery of scientific content	1: Participants’ response to training	Male	15	15	9
			Female	4	16	1
			Total	19(31.7%)	31(51.7%)	10(16.7%)
2	Please grade the adequacy of the simulated practical lab. in the achievement of the intended learning objectives	1: Participants’ response to training	Male	12	22	5
			Female	10	10	1
			Total	22(36.7%)	32(53.3%)	6(10%)
3	Please grade the capacity of the simulated practical lab. in helping the understanding of theoretical concepts	2: Participants’ learning from training	Male	13	19	7
			Female	8	10	3
			Total	21(35%)	29(48.3%)	10(16.7%)
Domain 2: Practical lab.						
			Sex	Yes	No	I do not Know
4	Did the simulated practical lab. yield realistic data that enable you to estimate the required parameters?	3: Participants' application of training	Male	24	6	9
			Female	13	6	2
			Total	37(61.7%)	12(20%)	11(18.3%)
5	Does the simulated practical lab. encourage formulating wet hands-on experiments to test the hypothesis?	2: Participants’ learning from training	Male	19	14	6
			Female	10	7	4
			Total	29(48.3%)	21(35%)	10(16.7%)
6	Do you want to repeat simulated practical labs in other disciplines?	4: Benefits to the institution	Male	22	13	4
			Female	5	12	4
			Total	27(45%)	25(41.7%)	8(13.3%)
			Sex	Long	Suitable	Short
7	Please rate the time needed to conduct the simulated practical lab.?	1: Participants’ response to training	Male	22	17	0
			Female	9	12	0
			Total	31(51.7%)	29(48.3%)	0(0.0%)

## Discussion

Research comparing simulated computer-based labs and traditional animal-based labs share a common basic study design. Students are divided into two groups: a control group learns using traditional animal-based methods while the test group learns using the newer or alternative method. Following the learning period, each group is evaluated using a standardized test that permits comparison between the two groups [6]. The superiority of the first method, in terms of cost and time-effectiveness, is reported by the determinations of investigators who followed up the above-mentioned study design [7-13]. Within Europe, shifting to alternative methods has become a legislative requirement according to the European Convention 123, which absolutely prohibits students’ use of animals in their basic university courses. The European Directive 86/609 advocates the use of alternatives wherever possible [14]. As part of enshrining the right to conscientiously object and the right to freedom of religion, it is becoming an institutional norm to indicate the use of animal experiments, if it is the case, to students at their application for admission [14]. The present study focused on the assessment of a simulated computer-based pharmacology lab. The assessment focused, among other attributes, on the ability of the method to reach the intended learning objectives and the provision of a good learning experience. Even though the assessment of learning methods, as distinct from the assessment of individual students, however, the two assessment levels closely overlap because student performance is the typical parameter by which a learning method is evaluated [14]. This is different from the validation of the method that necessitates a control to measure against the validated method, which we felt was unnecessary and old news. The present determinations of our students’ acceptance and good rating of the method are consistent with our hypothesis and with previous reports that students can experiment freely with these methods, learning both the subject matter and the scientific method itself in the process, and because they have more control over the practical class, they are able to engage in experiments repeating and/or problems solving until they know they have mastered knowledge and skill [14]. Female students’ determinations are significantly lower than male students. Similar observations on gender performance differences are reported and justified, by previous investigators [5]. These justifications included the wording of “male-oriented” questions or asking demographic questions before administering the test [5].

## Conclusions

An urge for the importance of shifting from “established practice” to “good science”, especially at the basic level of education, is signaled by the conclusions that simulated pharmacology labs provide an effective teaching method that satisfies all the criteria of the Kirkpatrick evaluation model. It yields realistic data that enable students to estimate the required pharmacological parameters. It provides a good learning experience that motivates students to formulate wet hands-on experiments and wish to repeat them in other disciplines.

## References

[1] Miller K, Lasry N, Chu K, Mazur E (2013). Role of physics lecture demonstrations in conceptual learning. Phys Rev ST Phys Educ Res.

[2] Hughes IE (2001). Do computer simulations of laboratory practicals meet learning needs?. Trends Pharmacol Sci.

[3] Scalise K, Timms M, Moorjani A, Clark L, Holtermann K, Irvin PS (2011). Student learning in science simulations design features that promote learning gains. J Res Sci Teach.

[4] Finkelstein ND, Adams WK, Keller CJ (2005). When learning about the real world is better done virtually: a study of substituting computer simulations for laboratory equipment. Phys Rev ST Phys Educ.

[5] Madsen A, McKagan SB, Sayre EC (2013). Gender gap on concept inventories in physics: what is consistent, what is inconsistent, and what factors influence the gap?. Phys Rev ST Phys Educ.

[6] Balcombe J (2006). Assessment of Alternatives in Education. From Guinea Pig to Computer Mouse. https://www.interniche.org/ru/system/files/public/Resources/Book/jukes_and_chiuia_-_2003_-_from_guinea_pig_to_computer_mouse_interniche_2nd_ed_en.pdf.

[7] Zbinden G, Flury-Roversi M (1981). Significance of the LD50-test for the toxicological evaluation of chemical substances. Arch Toxicol.

[8] Dewhurst DG, Meehan AS (1993). Evaluation of the use of computer simulations of experiments in teaching undergraduate students. British J Pharm Proc.

[9] Dewhurst DG, Jenkinson L (1995). The impact of computer-based alternatives on the use of animals in undergraduate teaching. Altern Lab Anim.

[10] Fawver AL, Branch CE, Trentham L, Robertson BT, Beckett SD (1990). A comparison of interactive videodisc instruction with live animal laboratories. Am J Physiol.

[11] Henman MC, Leach GDH (1983). An alternative method for pharmacology laboratory class instruction using biovideograph video tape recordings. Br J Pharmacol.

[12] Phelps JL, Nilsestuen JO, Hosemann S (1992). Assessment of effectiveness of videodisc replacement of a live-animal physiology laboratory. Distinguished Papers Monograph.

[13] Samsel RW, Schmidt GA, Hall JB, Wood LD, Shroff SG, Schumacker PT (1994). Cardiovascular physiology teaching: computer simulations vs. animal demonstrations. Am J Physiol.

[14] Jukes N, Chiuia M (2006). From Guinea Pig to Computer Mouse. Alternative Methods for a Progressive, Humane Education. https://www.interniche.org/ru/system/files/public/Resources/Book/jukes_and_chiuia_-_2003_-_from_guinea_pig_to_computer_mouse_interniche_2nd_ed_en.pdf.

